# Genetic Divergence across Habitats in the Widespread Coral *Seriatopora hystrix* and Its Associated *Symbiodinium*

**DOI:** 10.1371/journal.pone.0010871

**Published:** 2010-05-27

**Authors:** Pim Bongaerts, Cynthia Riginos, Tyrone Ridgway, Eugenia M. Sampayo, Madeleine J. H. van Oppen, Norbert Englebert, Francisca Vermeulen, Ove Hoegh-Guldberg

**Affiliations:** 1 Global Change Institute, The University of Queensland, St Lucia, Queensland, Australia; 2 Centre for Marine Studies, The University of Queensland, St Lucia, Queensland, Australia; 3 ARC Centre of Excellence for Coral Reef Studies, The University of Queensland, St Lucia, Queensland, Australia; 4 School of Biological Sciences, The University of Queensland, St Lucia, Queensland, Australia; 5 Climate Change Group, Great Barrier Reef Marine Park Authority, Townsville, Queensland, Australia; 6 Department of Biology, The Pennsylvania State University, University Park, Pennsylvania, United States of America; 7 Australian Institute of Marine Science, Townsville, Queensland, Australia; 8 ARC Centre of Excellence for Coral Reef Studies, James Cook University, Townsville, Queensland, Australia; 9 School of Biological Sciences, Victoria University of Wellington, Wellington, New Zealand; Northeastern University, United States of America

## Abstract

**Background:**

Coral reefs are hotspots of biodiversity, yet processes of diversification in these ecosystems are poorly understood. The environmental heterogeneity of coral reef environments could be an important contributor to diversification, however, evidence supporting ecological speciation in corals is sparse. Here, we present data from a widespread coral species that reveals a strong association of host and symbiont lineages with specific habitats, consistent with distinct, sympatric gene pools that are maintained through ecologically-based selection.

**Methodology/Principal Findings:**

Populations of a common brooding coral, *Seriatopora hystrix*, were sampled from three adjacent reef habitats (spanning a ∼30 m depth range) at three locations on the Great Barrier Reef (n = 336). The populations were assessed for genetic structure using a combination of mitochondrial (putative control region) and nuclear (three microsatellites) markers for the coral host, and the ITS2 region of the ribosomal DNA for the algal symbionts (*Symbiodinium*). Our results show concordant genetic partitioning of both the coral host and its symbionts across the different habitats, independent of sampling location.

**Conclusions/Significance:**

This study demonstrates that coral populations and their associated symbionts can be highly structured across habitats on a single reef. Coral populations from adjacent habitats were found to be genetically isolated from each other, whereas genetic similarity was maintained across similar habitat types at different locations. The most parsimonious explanation for the observed genetic partitioning across habitats is that adaptation to the local environment has caused ecological divergence of distinct genetic groups within *S. hystrix*.

## Introduction

The tropical marine realm harbors an incredible array of species, with coral reef ecosystems being the iconic epitome of this diversity. Classically, this diversity has been explained through allopatric models of speciation, in which reproductive isolation arises through the physical separation of populations [1]. However, speciation has also been demonstrated to occur sympatrically or parapatrically, where divergence originates in the absence of physical barriers and is driven by ecological sources of divergent selection (i.e. selection occurring in opposing directions) (reviewed in [2]). A classic marine example of incipient speciation in sympatry is that of the intertidal snail *Littorina saxatilis* comprising genetically distinct ecotypes (with different shell morphologies), which are partitioned over a gradient of tidal height [3]–[5]. Eventually, divergent selection can lead to complete reproductive isolation (i.e. ecological speciation), either as a by-product of divergent selection (via linkage disequilibrium [6]), or directly when the genes involved in reproductive isolation are under ecologically-based divergent selection (via pleiotropy [2]). Over the past decade, ecological speciation has been suggested as an explanation for the diversification of various terrestrial and freshwater taxa [7]–[12], yet only three examples have been proposed for tropical reef organisms: wrasses of the genus *Halichoeres* [13], sponges of the genus *Chondrilla*, [14], [15]), and the scleractinian coral *Favia fragum* [16].

Ecological diversification can arise through various sources of divergent selection (reviewed in [2]), including sexual selection (e.g. selection on mate recognition traits) and ecological interactions (e.g. interspecific competition). However, divergent selection between distinct environments is probably the best understood cause of ecological speciation [2] and has been proposed as a major contributing factor to the diversification of species in environmentally heterogeneous ecosystems such as tropical rainforests [7]. Coral reefs provide a similarly heterogeneous environment, with large variability in abiotic factors such as light [17], temperature [18], nutrient variability, and wave action [19], [20] between locations and also across depths at a single location. Although such environmental variability may favor a certain degree of plasticity [21], there is also the potential for locally adapted “ecotypes” to evolve through divergent selection [22]. Surprisingly, little is known about genetic structuring of coral reef populations across distinct habitats [23] and the potential role of environmental heterogeneity in species diversification.

Numerous observations indicate that ecological diversification may be important in coral evolution. Firstly, there are many examples where closely related, sympatric species occupy only part of the available habitat, i.e. they occupy a distinct environmental niche [24]–[26]. The Caribbean coral genus *Madracis* provides a good example in point; it consists of six closely related morpho-species [27], each of which has a distinct depth-distribution [28]. Other examples of closely related species exhibiting similar habitat partitioning are members of the genus *Agaricia* [29], [30], the *Montastraea annularis* species complex [31], [32], and the acroporids, *Acropora palifera* and *A. cuneata* [33], [34]. Secondly, on an intra-specific level, there are several observations suggestive of local adaptation (reviewed in [35]), such as local dominance of certain genets [36], [37] and variation between populations in thermal tolerance [38], and natural disease resistance [39]. Thirdly, many studies have observed cryptic diversity (e.g., in the genera *Seriatopora* [40], [41], *Pocillopora* [42], and *Porites* [43]) and genetic differentiation over small spatial scales in the absence of physical barriers (e.g., in population genetics studies on *Seriatopora* [44], [45]). Despite these lines of evidence, the specific hypothesis of ecological diversification remains largely untested for corals. Most genetic assessments have focused on concordance of observed patterns with morphology or geography rather than physiological characteristics or habitat. Exceptions are the studies by Carlon and Budd [16], which established that morpho-types of the coral *F. fragum* are genetically distinct and partitioned over a small depth gradient (∼3 m), and Ayre et al. [46], which demonstrated that a proportion (16%) of the genetic variability of *Seriatopora hystrix* within reefs could be explained by distributions among five shallow reef habitats (reef slope, reef crest, reef flat, lagoon, and back reef).

Habitat partitioning and ecological diversification have also been observed for the photosymbiotic partners (*Symbiodinium*) of scleractinian corals. Various coral species harbor distinct depth-specific symbiont types across their distribution range (e.g. [47]–[49]). Despite this apparent flexibility to associate with various symbiont types over depth [50], the coral-algal symbiosis is generally characterized by a high degree of host-symbiont specificity [51], in that coral species usually only associate with certain types of *Symbiodinium* and vice versa. This specificity is especially apparent in corals with a vertical symbiont transmission strategy, in which symbionts are passed directly from the maternal colony to the offspring [52]–[54]. Thus, corals with vertical symbiont transmission are most likely to codiversify with their algal symbionts but this process is poorly understood since studies considering both host and symbiont identity with a fine-scale genetic resolution are rare [53].

**Figure 1:**
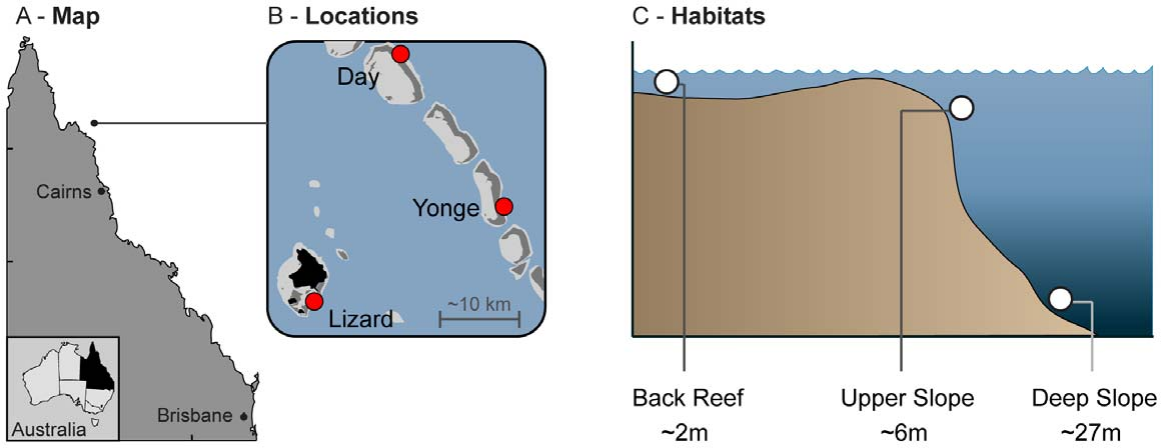
Sample design and locations. (A) Map showing the geographic location of the study area on the northern Great Barrier Reef; (B) the reef locations within the study area; and (C) the different habitats sampled.

The scleractinian coral *S. hystrix* represents an ideal candidate to examine processes of ecological diversification and local adaptation in corals, as it occurs in most habitats [55], and is geographically widespread [26]. *S. hystrix* exhibits a brooding reproductive strategy and vertically transmits associated *Symbiodinium*. Furthermore, *S. hystrix* has been the subject of several genetic studies [44]–[46], [56]–[60], with previous allozyme work indicating that genetic structuring of the coral host may occur among shallow habitats [46]. Here, we specifically test the extent of genetic structuring over a large depth range for both symbiotic partners, and evaluate genetic differentiation between the same habitat types at different locations. Focusing on three adjacent, environmentally distinct habitats (spanning a depth range of ∼30 m) at three locations (Yonge Reef, Day Reef and Lizard Island; Figure 1) on the northern Great Barrier Reef, we used a combination of mitochondrial (putative control region) and nuclear (microsatellites) markers for the host, and the ITS2 region of the nuclear ribosomal DNA for *Symbiodinium* to assess genetic differentiation. Results indicate that adjacent habitats within a single reef can be genetically isolated from each other, whereas genetic similarity is maintained between the same habitat types at different locations. The strong partitioning of both host and symbiont lineages occurs between directly adjacent habitats in the absence of physical dispersal barriers, and thus provides a compelling case for divergence due to ecologically-based divergent selection.

## Results

**Figure 2:**
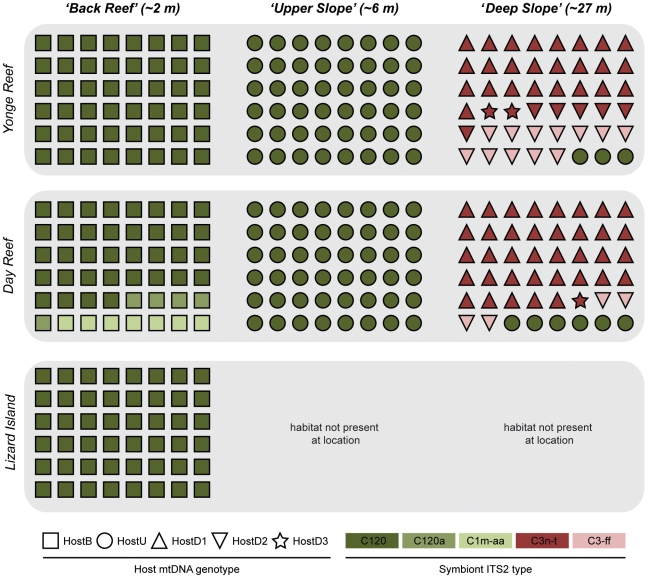
Diversity and distribution of host genotypes (mtDNA) and symbiont lineages (ITS2) across the three habitats and locations. Symbols refer to individual coral colonies, with the symbol shape indicating host genotype and symbol color indicating symbiont genotype. Shaded boxes group the different habitats at a reef locality.

**Figure 3:**
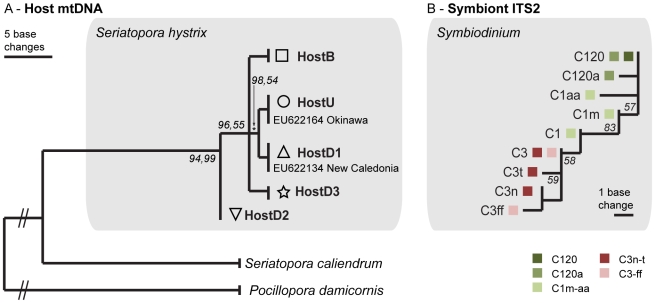
Phylogenetic trees of *Seriatopora hystrix* and associated *Symbiodinium* lineages. (A) Phylogenetic tree (maximum likelihood) of *S. hystrix* mitochondrial haplotypes, with *Seriatopora caliendrum* and *Pocillopora damicorni*s as outgroups. Bootstrap values (in italics) are based on Bayesian analyses and ML respectively, with only probabilities over 50% shown. The shaded box groups the various *S. hystrix* haplotypes observed in this study. The ‘HostU’ and ‘HostD1’ haplotypes match with previously obtained Genbank sequences. (B) Unrooted phylogenetic tree (maximum parsimony) of the five distinct *Symbiodinium* types. Colors group ITS2 sequences belonging to a single *Symbiodinium* type (for example *Symbiodinium* type C120 contains only the ITS2 sequence C120 while *Symbiodnium* C120a contains both the C120 and C120a sequence within its genome). Bootstrap values higher than 50% are shown in italics.

Strong genetic structuring of both coral host and the associated *Symbiodinium* was observed across the different reef habitats (*‘Back Reef’* ∼2 m, *‘Upper Slope’* ∼6 m and *‘Deep Slope’* ∼27 m) and the results were consistent across all investigated loci. In contrast, little to no genetic divergence was found between similar habitats of Yonge Reef and Day Reef, positioned at the edge of the continental shelf on the GBR, and Lizard Island, located mid-shelf (Figures 1, 2). Additionally, there was strong coupling of host and symbiont genotypes (Figures 2, 3).

### Coral host - mtDNA

Analyses of the putative control region of the coral mtDNA indicated strong genetic partitioning between the three different habitat types. In contrast, there was no differentiation among the same habitat types at different locations (Figure 2). The mtDNA region (557–608 bp) contained 10 variable sites as well as an indel, defining a total of 5 different haplotypes (Figures 2, 3; GenBank HM159623-HM159958). All colonies from the *‘Back Reef’* habitat at Yonge Reef, Day Reef, and Lizard Island shared a single host mtDNA haplotype, ‘HostB’ (n = 144). All colonies from the *‘Upper Slope’* habitat at Yonge Reef and Day Reef shared a single host mtDNA haplotype, ‘HostU’ (n = 96), that differed from the *‘Back Reef’* haplotype ‘HostB’ by 4 substitutions. The *‘Deep Slope’* habitat harbored three additional mtDNA haplotypes: ‘HostD1’ (n = 62), ‘HostD2’ (n = 22) and ‘HostD3’ (n = 3); as well as a few ‘HostU’ genotypes (n = 9). The genotypic community structure was significantly different (Two-Way ANOSIM; habitat nested within location) between habitat types (R = 0.804; p = 0.001), but not reef locations. Under the AMOVA framework, 83% (Φ_HAB-TOT_ = 0.832; p = 0.01) of molecular variance was explained by habitat, and only 2% by location (Φ_LOC-HAB_ = 0.129; p = 0.01).

Phylogenetic analyses (MP and Bayesian analyses) of the putative control region, using *Seriatopora caliendrum* (Genbank EF633600) and *Pocillopora damicornis* (Genbank NC009797) as outgroups, supported the monophyly of *S. hystrix* with high bootstrap support (Figure 3). The *S. hystrix* ‘HostD2’ genotype represents the most likely ancestor of the five mtDNA genotypes (95% Bayesian posterior probability; MP bootstrap support of 55%; Figure 3). The other three genotypes are more recently diverged with a distinct lineage grouping the ‘HostU’ and ‘HostD1’ genotypes separately from the ‘HostB’ and ‘HostD3’ genotypes (98% Bayesian posterior probability; MP bootstrap support of 54%; Figure 3). The shallow host genotype ‘HostU’ contained a 51 bp tandem repeat (excluded from the phylogenetic analysis), and was identical to previously obtained *S. hystrix* sequences from Taiwan and the South China Sea [41] (Genbank EF633596-EF633599, EF633584-EF633589) as well as Okinawa [40] (‘Cluster 2’; Genbank EU622164-EU622165) (note that for the reference sequences no habitat information was available). Additionally, the deep host haplotype ‘HostD1’ was identical to observed haplotypes of *S. hystrix* collected from depths between ∼7–40 m around New Caledonia [40] (‘Cluster 1’; Genbank EU622134, EU622151, EU622159- EU622163). Despite the genetic differences among the five haplotypes being small (2-5 bp) compared to the sister species *S. caliendrum* (29 bp - excluding indels), individual haplotypes corresponded with habitat type and associated symbiont rather than location, even on a broader geographical scale.

### Coral host - microsatellites

Analyses of three nuclear (microsatellite) loci corroborated the differentiation suggested by the mtDNA and revealed a similar pattern of habitat partitioning. A total of 172 unique multilocus genotypes were observed among 200 analyzed samples. Of these multilocus genotypes, 17 were shared between 2 individuals, 4 between 3 individuals, and 1 between 4 individuals. With the exception of one individual, these potential clone mates always occurred within the same habitat and location, and may have resulted from fragmentation.

**Figure 4:**
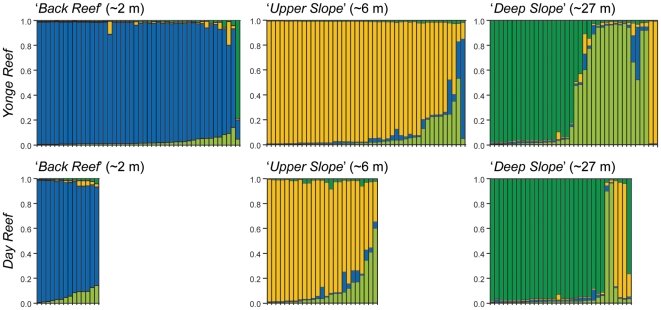
Subdivision of *Seriatopora hystrix* populations as inferred by microsatellite loci using STRUCTURE (K = 4). Analyses were run with no *a priori* information assumed on sample origin. The probability of assigning each individual coral (on the x-axes) to one of the four clusters (represented by the four colors) is shown on the y-axes. The different clusters correspond largely with the mtDNA haplotypes: ‘HostB’ (blue cluster), ‘HostU’ (yellow cluster), ‘HostD1’ (dark-green cluster) and ‘HostD2’/’HostD3’ (light-green cluster).

Genetic clustering was first assessed using STRUCTURE v2.2 [61] without providing *a priori* population designations. Analyses were done with and without clonal multilocus genotypes, and rendered near identical patterns of clustering and log probability distributions. The highest log probability was found for K = 4 (i.e. 4 genetic clusters; Table S1), which divided the dataset into four clusters that strongly corresponded with the four common mtDNA haplotypes (‘HostB’, ‘HostU’, ‘HostD1’ and ‘HostD2’) of each sample (Figure 4). As a consequence, all individuals with mitochondrial genotype ‘HostB’ sampled from the *‘Back Reef’* habitat formed a single cluster (regardless of location), as did all the individuals from the *‘Upper Slope’* habitat with mtDNA genotype ‘HostU’. Individuals sampled in the ‘*Deep Slope’* habitat with a ‘HostU’ haplotype (n = 6) were assigned to the same group as the *‘Upper Slope’* individuals with the ‘HostU’ genotype (with one exception). All remaining ‘*Deep Slope’* samples clustered in two groups corresponding to the haplotypes ‘HostD1’ and ‘HostD2’. The two individuals with a ‘HostD3’ genotype clustered together with ‘HostD2’ individuals (despite the presence of a private allele in these samples). A slightly lower log probability was found for K = 3 (Table S1), which resulted in an identical clustering by habitat, but without further subdivision of individuals from the *‘Deep Slope’* habitat (i.e. individuals with a ‘HostD1’ and ‘HostD2’ mtDNA genotype form each a single cluster).

To avoid a Wahlund effect (sampling across distinct genetic cohorts) during further analysis under the AMOVA framework, the *‘Deep Slope’* habitats were reduced to individuals with a ‘HostD1’ genotype. This was done as STRUCTURE results indicated that the other haplotypes in this habitat (‘HostD2’, ‘HostD3’, and ‘HostU’) belong to different genetic clusters (Figure 4). Clone mates in the same population were also removed before analyses, so that multi-locus genotypes only occurred once. A nested AMOVA of the resulting populations indicated that 18% of molecular variance was partitioned between habitats (Φ_HAB-TOT_ = 0.187; p<0.001), as opposed to 5% between locations (Φ_LOC-HAB_ = 0.066; p<0.001). Pairwise F_ST_ values of populations (with the ‘HostD2’ individuals at Yonge Reef as a seventh population) corroborated this strong differentiation between habitats (F_ST_ = 0.179–0.300), rather than among habitats at different locations (F_ST_ = 0.012 and 0.060; Table 1). An exception was the level of differentiation observed between the *‘Back Reef’* habitats at Day and Yonge Reefs (F_ST_ = 0.120), however this genetic structuring was still less than any of the across-habitat pairwise differences (F_ST_ = 0.201–0.275). Finally, the subclusters of the *‘Deep Slope’* habitat at Yonge Reef with ‘HostD1’ and ‘HostD2’ haplotypes respectively, were also genetically distinct (F_ST_ = 0.179). Linkage disequilibrium (LD) was not detected for any of the loci or population pairwise comparisons. Significant deviation from Hardy-Weinberg equilibrium (HWE) was detected only in the *‘Upper Slope’* habitat at Yonge Reef for the locus Sh2-006, whereas all three loci were in HWE in the remaining populations (Table S2). Estimates of inbreeding for the different populations were consistently low (F_IS_ = 0.0063–0.0793), again with exception of the *‘Upper Slope’* habitat at Yonge Reef (F_IS_ = 0.1342; Table S2).

**Table 1:** Pairwise estimates of F_st_ values from three microsatellite loci for the coral *Seriatopora hystrix*.

		‘*Back Reef*’		‘*Upper Slope*’		‘*Deep Slope*’	
		Day	Yonge	Day	Yonge	Day	Yonge
**‘** ***Back Reef*** **’**	**Day**						
	**Yonge**	**0.120** **					
**‘** ***Upper Slope*** **’**	**Day**	0.275**	0.228**				
	**Yonge**	0.244**	0.206**	**0.060** *			
**‘** ***Deep Slope*** **’**	**Day**	0.300**	0.255**	0.246**	0.288**		
	**Yonge**	0.253**	0.213**	0.219**	0.241**	**0.012** NS	
**‘** ***Deep Slope*** **’**	**Day**	0.184**	0.146**	0.224**	0.201**	0.179**	0.199**
**(‘HostD2’)**							

Comparisons were calculated between three different habitats (‘*Back Reef*’, ‘*Upper Slope*’, ‘*Deep Slope*’) at two locations on the northern GBR (Day Reef, Yonge Reef). The last population consists of the individuals from the ‘*Deep Slope*’ habitat at Yonge Reef with a ‘HostD2’ mtDNA genotype. Values in bold indicate pairwise values between the same habitats at different locations.

NS = Not significant,

*p = 0.001 and

**p<0.001.

### *Symbiodinium* - ITS2

Based on the ITS2 rDNA region, 5 distinct symbiont types were found in association with the *S. hystrix* colonies sampled (Figure 2). The symbiont profiles consisted of the previously described *Symbiodinium* C3n-t [48], and four novel symbiont types belonging to clade C (Figure 2; C120, C120a, C1m-aa, C3-ff). Four of the five *Symbiodinium* types (except C120) contained 2-3 co-dominant ITS2 sequences within a single profile (see ITS2-DGGE profiles in Figure S1; Genbank HM185737-HM185741). Sequences within a profile differed by only 1–2 bases and all ITS2-DGGE fingerprints were highly consistent across samples. As such, sequences present within a single profile were considered co-dominant rDNA repeats (i.e. intragenomic variants) within the genome of a single *Symbiodinium* type [52], [62].

Specific symbiont types were found in association with *S. hystrix* colonies from either shallow (*‘Back Reef’* and *‘Upper Slope’*) or deep habitats (*‘Deep Slope’*). Approximately 95% of the colonies collected from the shallow habitats (*‘Back Reef’* and *‘Upper Slope’*) harbored *Symbiodinium* C120 irrespective of location (Yonge Reef, Day Reef, and Lizard Island), with a few *‘Back Reef’* habitat colonies at Day Reef harboring *Symbiodinium* C120a and C1m-aa (5 and 7 colonies respectively). In contrast, the majority (74%) of colonies from the *‘Deep Slope’* at Yonge Reef and Day Reef hosted *Symbiodinium* C3n-t (n = 69). The remaining 26% of *‘Deep Slope’* colonies harbored *Symbiodinium* C3-ff (n = 16) or *Symbiodinium* C120 (n = 9). Symbiont diversity proved significantly different (Two-Way ANOSIM; habitat nested within location) between habitats (R = 0.444; p = 0.001), but not locations. Pairwise comparisons between the various habitat types (One-Way ANOSIM) confirm that the *‘Deep Slope’* habitat is significantly different from *‘Back Reef’* (R = 0.676; p = 0.001) and *‘Upper Slope’* habitat (R = 0.696; p = 0.001), but that the difference between *‘Back Reef’* and *‘Upper Slope’* is not significant.

Phylogenetic analysis indicated that the shallow *Symbiodinium* types C120, C120a, and C1m-aa were more closely related to each other than to the ‘deep types’ C3n-t and C3-ff. The shallow *Symbiodinium* types appeared to have evolved from the ancestral type C1 while the deep *Symbiodinium* types C3n-t and C3-ff are diverged from C3 (and still contain the C3 sequence within their ribosomal array) [62].

### Coupling of host and symbiont genotypes

The overlay of the *Symbiodinium* ITS2 types over the host mtDNA data (Figure 2) showed a strong association between host and symbiont lineages (Figure 3). All *S. hystrix* colonies with haplotype ‘HostD1’ and ‘HostU’ exclusively harbored *Symbiodinium* C3n-t and C120 respectively. Individuals with haplotype ‘HostD2’ harbored *Symbiodinium* C3-ff (73%) and C3n-t (27%). Similarly, most colonies with haplotype ‘HostB’ harbored *Symbiodinium* C120 (92%), but C120a (3%) and C1m-aa (5%) were also observed in low numbers. There was a significant difference (One-way ANOSIM) between the types of *Symbiodinium* associated with each mtDNA haplotype (R = 0.528; p = 0.001) as well as the types of mtDNA haplotypes each *Symbiodinium* type associated with (R = 0.454; p = 0.001). A further indication of the strong association between host mtDNA and symbiont ITS2 genotypes were the individuals with the shallow water ‘HostU’ haplotype that were found in the deep environment and always contained *Symbiodinium* C120, common among shallow water colonies.

## Discussion

This study demonstrates that *S. hystrix* and its associated *Symbiodinium* form genetically isolated clusters across distinct reef habitats (Figures 2,3,4). The association of host lineages (mtDNA) and genetic clusters (nDNA) with particular reef-environments rather than geographic location is consistent with divergence occurring through ecologically-based selection. Furthermore, the observed coupling of host and symbiont genotypes points to codiversification at a fine taxonomical level.

### Habitat partitioning of coral host populations/genotypes

The three habitats sampled in this study (*‘Back Reef’*, *‘Upper Slope’*, *‘Deep Slope’*; Figure 1) differ greatly in exposure to wave action, temperature regimes and light availability (see methodsfor detailed description). Across these habitats, strong partitioning of host mtDNA haplotypes is observed, with the *‘Back Reef’* and *‘Upper Slope’* habitats each containing a single haplotype and the *‘Deep Slope’* habitat containing four different haplotypes. Similarly, Bayesian analysis of three microsatellite loci revealed four genetic clusters, with each cluster corresponding to one of the four common mtDNA haplotypes (i.e., individuals in each cluster share the same mtDNA genotype). There was also strong genetic differentiation across habitats based on microsatellites under the AMOVA framework. Replication of these striking genetic patterns across two distinct reef locations (∼20 km apart) is consistent with local adaptation to distinct habitats followed by non-random mating, as the genetic structure was observed in putative neutral loci. Detectable genetic structure based on linkage disequilibria among microsatellite loci can develop over relatively short timescales, however partitioning based on mitochondrial loci should reflect longer (evolutionary) timescales. As such, the observed partitioning likely reflects long-standing adaptations to the unique environmental conditions of each habitat, such as strong wave action (e.g. *‘Upper Slope’*), extreme temperature fluctuations (e.g. *‘Back Reef’*) or low-light conditions and cold-water influxes (e.g. *‘Deep Slope’*). However, given that various abiotic factors covary between habitats, it is impossible at this time to assess the likely contribution of specific environmental variables to the observed genetic partitioning. Although diversity generally declines with depth in coral species [63] and *Symbiodinium* types [48], [62], here we observed the highest diversity of host and symbiont genotypes in the deeper habitat.

Even though populations of *S. hystrix* are generally highly structured geographically across the Great Barrier Reef (GBR) [45], [46], [56], [58], van Oppen et al. [45] reported high genetic similarity between populations on the Ribbon Reefs (including Yonge Reef) of the northern GBR with pairwise F_ST_ values ranging from 0.009–0.026 for reefs up to ∼80 km apart. Additionally, genetic similarity was observed between the Ribbon Reefs and a population at Lizard Island (F_ST_ = 0.065–0.090). These results are concordant with the genetic similarity observed in this study between Yonge Reef and Day Reef (within habitats) using a subset of the microsatellite loci used by van Oppen et al. [45] (F_ST_ = 0.012–0.060) and between Yonge Reef, Day Reef and Lizard Island (within habitats) for the mtDNA locus. Thus, despite the highly localized recruitment of *S. hystrix* [44], larval exchange between directly adjacent habitats (∼50–500 m apart) is unlikely to be hampered by physical barriers. Rather, the differentiation across habitats seems to be driven by non-allopatric diversification processes.

Ayre and Dufty [46] were the first to identify an effect of habitat on genetic differentiation in *S. hystrix*. In their allozyme study they reported that a proportion of the within reef variability was explained by variation among five shallow habitat types (F_HR_ = 0.05) on the central GBR. A later study by Sherman [60] at a single location on the southern GBR reported little differentiation between habitats (F_HR_ = 0.009), but did find different levels of inbreeding between habitats (also observed by Ayre and Dufty [46]). The study by van Oppen et al. [45] did not specifically assess differences between habitats, but they did report one population in the Ribbon Reefs (which was sampled at a different depth and during a different year compared to the other populations) that was highly divergent from the other Ribbon Reef populations, leading them to suggest that this was either a reflection of temporal variability or was driven by habitat. A similar pattern of differentiation was found for a population sampled on the exposed side of Davies Reef [45]. In this study, we reconfirm the effect of habitat on genetic differentiation, first detected by Ayre and Dufty [46], but also demonstrate that the extent of differentiation between adjacent habitats can entail fixed differences.

Significant F_IS_ and genetic structuring within populations are commonly observed among corals and have previously been attributed to local inbreeding or Wahlund effects [44]. High levels of inbreeding were only detected in the *‘Upper Slope’* habitat at Yonge Reef, possibly due to the lower densities of *Seriatopora* colonies in the *‘Upper Slope’* habitat (Bongaerts et al. unpublished data). In contrast to most previous studies [44 and references therein], allele frequencies in all other populations approached expectations under HWE. Although local mating would lead to inbreeding, it would not create the replicated associations (at two different reefs) of genotypes and habitats that we observed. By sampling within distinct habitats we seem to have avoided any sign of a Wahlund effect in our data, which may have affected previous studies if distinct ecotypes were present in sample locations. The exception in our study is the *‘Deep Slope’* habitat that does contain multiple distinct genetic groups (Figures 2, 4).

### Depth zonation of *Symbiodinium*

The observed partitioning of *Symbiodinium* types across habitats (Figure 2) matches numerous reports on symbiont zonation over depth (e.g. [47]–[49], [62], [64]) and could reflect adaptation to depth-related environmental conditions such as low-light conditions [65]–[67]. Although some overlap existed, the common shallow symbiont, *Symbiodinium* C120, was rarely encountered in the *‘Deep Slope’* habitat and neither of the deep symbiont types, C3n-t or C3-ff, were found in the shallow habitats. This zonation of symbionts in *S. hystrix* differs from results on the southern GBR, where *Symbiodinium* C3n-t was found to occur in colonies from 3 to 18 meters depth [48]. The differences in depth range of *Symbiodinium* C3n-t between these studies (southern GBR, depth generalist; northern GBR, deep specialist) may reflect latitudinal variation in surface irradiance and light attenuation (with lower irradiance levels recorded on the southern GBR [68]). However, various abiotic factors other than light change with increasing depth (e.g. spectral quality, temperature, nutrient availability), and as with the host, it is therefore difficult to assess the individual contribution of each factor to the observed partitioning of symbionts [49]. Alternatively, different host-symbiont associations may predominate at different latitudes on the GBR.

### Codiversification in the coral-algal symbiosis

The coral-algal symbiosis has received much attention over the past decade (reviewed in [50], [69]), and host-symbiont specificity and stability are tightly linked to the ability of corals to respond to environmental change (i.e. the ability to change symbiotic partners as a mechanism to cope with change). Whereas many studies have focused on the genetic identity of the symbiont, this study is one of the few to evaluate host-symbiont specificity using molecular markers for both symbiotic partners [70]–[72]. The most striking finding was the habitat partitioning of linked symbiotic partners, which suggests adaptation of the holobiont (host plus symbiont) to distinct environmental niches and/or linkage disequilibrium on a genomic level. The *‘Upper Slope’* and *‘Back Reef’* host mtDNA genotypes (‘HostU’ and ‘HostB’) were found in symbiosis with two closely related shallow symbionts types, C120 and C120a, as well as the rare *‘Back Reef’* symbiont C1m-aa. The two common host genotypes associated with the *‘Deep Slope’* habitat occurred with *Symbiodinium* types C3n-t and C3-ff (Figure 2), with C3-ff occurring exclusively in individuals with the ‘HostD2’ genotype. The observed correlation reinforces that high levels of specificity occur, even among closely related host species [48], [49], [54], [73] and potentially at an intra-specific level (Figures 2,3). As such, our data underlines the potential importance of co-speciation processes in the diversification of both symbiotic partners, and this may be particularly important in corals with a vertical symbiont acquisition mode such as the brooding coral *S. hystrix* [52], [54], [73], [74].

It is noteworthy that in the few instances where holobiont genotypes seem ‘misplaced’ with regards to habitat, the host-symbiont genotype associations were maintained with reference to each other. For example, individuals sampled in the *‘Deep Slope’* habitat with the common *‘Upper Slope’* host genotype ’HostU’ (mtDNA) also contained the shallow symbiont C120 instead of any of the deep symbionts (Figures 2,4). These colonies may therefore be occurring near the lower depth limit of the ‘shallow’ population, and the *‘Deep Slope’* habitat may be encompassing a contact zone with mixed environmental conditions [13] that marks a transition from ‘shallow’ to ‘deep’ haplotypes. The observation of holobiont-habitat ‘mismatches’ reinforces the status of the mtDNA haplotypes as distinct host lineages. These lineages probably represent ecotypes or potentially incipient/cryptic species that differ in their depth distribution and symbiont types, thus phenotypic plasticity alone is unlikely to be the only mechanism by which *S. hystrix* can thrive under a broad range of environmental conditions.

### Ecological speciation

Ecological speciation describes a process of diversification that can occur in the absence of extrinsic barriers, and has therefore been proposed as an alternative to allopatric speciation in the tropical marine realm [13]. In many instances, ecological speciation is driven by divergent selection between environments and eventually results in habitat partitioning between closely related lineages. However, as divergent selection between environments is equally consistent with allopatric speciation [75], it is important to identify the geographic context in which speciation has occurred. Coyne and Orr [76] argue that divergence in sympatry must be demonstrated through a present-day sympatric distribution of the most closely related sister species and an ecological setting in which allopatric differentiation is unlikely. Yet, excluding any scenario of historical allopatry is impossible for most taxa [76], so that the most convincing cases of sympatric speciation have been limited to unique isolated terrestrial and freshwater settings, such as a crater lake [77] and a remote oceanic island [78].

In the coral reef environment, ecological speciation has been suggested in a few instances, where the general expectation of genetic partitioning according to habitat rather than biogeographical barriers was met. For example, Rocha et al. [13] observed strong genetic differentiation of several congeneric species of tropical reef fish (genus *Halichoeres*) across habitats, but not geographic locations. Similarly, Duran and Rützler [14] found partitioning of mtDNA haplotypes of a Caribbean marine sponge (genus *Chondrilla*) across mangrove and reef habitats, but not geographically distant locations. On a more local scale, Carlon and Budd [16] identified distinct depth distributions of *Favia fragum* morpho-types across three sites (up to ∼2 km apart) in the Bocas del Toro region (Panama), consistent with a ‘divergence with gene flow’ model [16]. In a similar fashion, we observe strong genetic segregation of the coral *S. hystrix* across environmentally distinct habitats, but not between the same habitats at different locations (∼20 km apart; Figure 2). As gene flow does not seem to be limited by physical barriers, the observed partitioning of *S. hystrix* in this study supports the notion of reduced gene flow through divergent selection between distinct reef habitats. Due to the limited geographic range and small number of sampled reefs, however, it is unclear whether the observed patterns of genetic differentiation are part of a broad-scale pattern in *S. hystrix*. Two of our mtDNA haplotypes match published *S. hystrix* sequences from other localities in the Indo-Pacific (Okinawa, New Caledonia, Taiwan) [40], [41] (Figure 3), suggesting a widespread occurrence of these lineages. Furthermore, as sampling was performed in discrete habitats rather than over a bathymetric gradient, it is unclear whether the observed partitioning reflects a step function (i.e. microallopatry) or distributions with zones of overlap. Further studies across a bathymetric gradient covering a broad geographic range could provide insights into the specifics and geographical context of the observed partitioning. Additionally, future studies should test whether genetic segregation is maintained through mainly pre- or post-settlement processes (e.g., reproductive isolation or selection against ecotypes that settle in the ‘wrong’ habitat).

Morphological features were not characterized, but gross morphology was observed to vary between habitats, similar to descriptions of ecotypes described by Veron and Pichot [55]. Colonies of *S. hystrix* in the *‘Back Reef’* and especially the *‘Upper Slope’* habitats seemed to have thicker branches (perhaps related to the greater extent of wave action in these habitats) in comparison to *‘Deep Slope’* individuals. Additionally, colonies in the *‘Upper Slope’* habitat were more compacted with shorter and more frequently dividing branches. Previous work by Flot et al. [34] in Okinawa, New Caledonia and the Philippines showed little congruence between mitochondrial sequences and morphological species delimitations, however they focused on genetic variability between various *Seriatopora* spp. (*S. hystrix*, *S. caliendrum*, *S. aculeata*, *S. guttatus,* and *S. stellata*; the latter three are not reported for the GBR) and specifically report distinct genetic lineages within *S. hystrix.* Although the taxonomic status of the observed mtDNA lineages will need to be resolved in future molecular and morphological studies (in order to assess whether they represent intra-specific diversity, subspecies (ecotypes), or cryptic species [40], [41]), at present, incipient ecological speciation seems to provide the most parsimonious explanation for the strong association of closely-related, sympatrically-occurring host lineages with habitat.

### Conclusions

Even though genetic variability between habitats has been previously demonstrated [46], this study clearly indicates that habitats within a reef can be genetically isolated from each other, whereas the same habitat types separated by up to ∼20 km can exhibit high levels of genetic similarity. Furthermore, the observed genetic partitioning demonstrates that the cryptic diversity previously detected in *S. hystrix* [40], [41] may be a reflection of lineages associated with distinct reef environments. Habitat-associated cryptic diversity may explain some of the “stochastic” results and high levels of genetic structuring over short geographic distances commonly observed in genetic studies of scleractinian corals. This study highlights the need to further explore genetic diversity over environmental gradients in other coral species, preferably encompassing species with a variety of life history strategies and broad ecological distributions. This is particularly important in the context of local reef connectivity and the general conception that deeper sections of reefs [18], [79] may act as a reproductive source for shallow reef areas following disturbance [23].

The strong association of host and symbiont genotypes with particular reef environments presents a compelling case for ecological speciation, corroborating previous evidence [13]–[15] that ecologically-based divergent selection may be an important mechanism for diversification on coral reefs. Overall, it underscores the need for understanding processes that shape diversity, which will allow for more accurate predictions on the persistence and community structure of coral reefs in a future of increasing anthropogenic and climate pressures.

## Materials and Methods

Collection of the corals was in accordance with the Queensland Animal Care and Protection Act 2001 and the necessary permits were supplied by the Great Barrier Reef Marine Park Authority (Townsville, Australia).

### Sample collection and processing

Small fragments (±3 cm) of *S. hystrix* colonies were collected (n = 336) on SCUBA from three different habitats: the *‘Back Reef’* (2 m depth ±1 m), *‘Upper Slope’* (6 m depth, ±1 m) and *‘Deep Slope’* (27 m depth ±2 m) at two reef locations, Yonge Reef (14°36′59.9″S; 145°38′ 11.1″E) and Day Reef (14°28′28.4″S/145°32′19.1″E) along the continental shelf edge of the GBR and from a *‘Back Reef’* habitat (2 m) at Lizard Island (14°41′39.1″S; 145°27′ 58.2″E). The three reef locations are at an approximate distance of 19–25 km from each other (Figure 1).

The *‘Back Reef’* habitat (∼2 m) is a shallow water body with strong temperature fluctuations during slack tides when there is little exchange with surrounding waters. The *‘Upper Slope’* (∼6 m) while not experiencing the same temperature fluctuations, faces the Coral Sea and experiences strong wave action from the incoming waves that break onto the reef (Done 1982) as it is located just below the reef crest. The *‘Deep Slope’* (∼27 m) on the other hand, has low irradiance levels compared to the shallow habitats and during summer, experiences slightly lower average temperatures (monthly average during February 2008 was ∼1°C lower) due to influxes of deep, sub-thermocline water (Bongaerts et al. unpublished data). Based on light attenuation data of the adjacent Ribbon Reefs, the proportion of surface irradiance available in the *‘Deep Slope’* habitat is also expected to be up to 10 times lower than in the shallower habitats (based on a K_g_ [PAR] of 0.084 during the summer solstice period; Veal, unpublished data;). Thus, despite their proximity (∼50 m between *‘Upper Slope’* and *‘Deep Slope’* habitats; ∼500 m between slope and *‘Back Reef’* habitats), these habitats offer very distinct environmental conditions to both the coral host and their associated photosymbionts.

Corals were identified as *S. hystrix* based on characters described by Veron [26] and Veron and Pichot [55]. All collected colonies were separated by at least 3 m in order to minimize the inclusion of potential clone mates due to fragmentation. Coral tissue was separated from the coral skeleton with a modified airgun attached to a SCUBA cylinder and subsequently stored in 20% DMSO preservation buffer and kept at −20°C until further processing. DNA (from both coral and symbiont) was extracted from the tissue using a Qiagen Plant Mini extraction kit following the manufacturer's instructions.

### Coral host - mtDNA genotyping

A fragment of the putative control region (atp6-nad4 intergenic spacer; [41]) was amplified for all samples (n = 336) using the newly designed primers SerCtl-F1: 5′-GTC TGC TCA CAT TAA TTT AT-3′ and SerCtl-R1: 5′-AGA GAT CGA ACT AAG AGT CG-3′. Primers were designed from the published *Seriatopora* mitogenome (Genbank Accession Number NC010244). PCR amplifications were performed with 0.1–1.0 µl of DNA, 2 µl 10x PCR buffer (Invitrogen), 1.0 µl 50 mM MgCl2, 1 µl 10 mM dNTPs, 1 µl 10 mM SerCtl-F1, 1 µl 10 mM SerCtl-R1, 0.10 µl of Platinum *Taq* DNA Polymerase (Invitrogen) and dH_2_0 water to a total volume of 20 µl per reaction. The cycling protocol was: 1×94°C (10 min); 30×[45 s at 94°C, 45 s at 56°C, 30 s at 72°C]; 1×72°C (8 min). PCR reactions were purified (using ethanol and ammonium acetate) and sequenced using both forward and reverse, or just the reverse primer (ABI BigDye Terminator chemistry, Australian Genome Research Facility). All chromatograms were checked manually and the resulting sequences aligned with *Seriatopora caliendrum* (Genbank No EF633600) and *Pocillopora damicornis* (Genbank No EF526302.1), which were subsequently used as outgroups. Modeltest [80] found that a HKY model [81] of molecular evolution (which allows for different rates of transition and transversion of the four nucleotides) best described the data under a log likelihood optimality criterion. Genealogies were constructed using maximum parsimony (MP) in the software PAUP* 4.0b10 [82] with indels excluded from the phylogenetic analysis. Bootstrapping was performed using a parsimony criterion (1000 replicates). Bayesian analyses were performed using the program MrBayes [83], with the Markov Chain Monte Carlo search run under the following conditions: 4 chains, 10^6^ generations, a sample frequency of 100 generations and a “burn-in” of 2500 trees. The individual contributions of habitat and location on genotypic variability were assessed under the AMOVA framework using GenALEx V6 [84], with location nested within habitat. Tests for statistical significance were based on 9,999 random permutations, followed by sequential Bonferroni correction.

### Coral host - microsatellite genotyping

Three polymorphic microsatellite loci (Sh4-001, Sh2-002, Sh2-006; [85]) were amplified for a subset of samples (n = 200) from Yonge Reef and Day Reef to verify whether nuclear loci corroborated the observed partitioning of mtDNA haplotypes (the *‘Back Reef’* habitat from Lizard Island was not included). Amplification was carried out by PCR incorporating a universal fluorescently labeled M13 primer following Schuelke [86] or by directly labeling the microsatellite primers following Underwood et al. [85]. The products were analyzed on a MegaBACE 1000 capillary sequencer (Amersham Biosciences) against an internal size-standard (ET 400-R, GE Healthcare) and the resulting electropherograms were scored using the program MegaBACE Genetic Profiler Version 2.2 (Amersham Biosciences). Genetic structuring in the dataset was explored using the Bayesian clustering method STRUCTURE v2.2 [61], which can assign individuals to genetic clusters without taking into account sample origin. The most likely number of genetic clusters (K) was inferred using the method of Evanno et al. [87], where both the log probability and the rate of change in log probability are considered. Five independent chains were run with a burn-in length of 100,000 and 1,000,000 MCMC replications (after burn-in) for K = 2 to K = 12, under an admixture model with independent allele frequencies and without *a priori* information about populations (as outlined in [45]). Genetic structuring was further assessed under the AMOVA framework using GenAlEx V6 [84], by partitioning the amount of genetic variation (with regards to alleles) between and within habitats (location nested within habitat). In this analysis, populations were reduced to individuals from the single, most dominant genetic cluster in that habitat (as indicated by the STRUCTURE results). Pairwise genetic distances (*F_ST_* values) between habitats at the various locations were also calculated. Tests for statistical significance were based on 9,999 random permutations, followed by sequential Bonferroni correction. Linkage disequilibrium and significant deviations from HWE were evaluated using GENEPOP (web version 3.4) [88].

### *Symbiodinium*–ITS2 genotyping

The internal transcribed spacer (ITS2) region of the rDNA for *Symbiodinium* was amplified for all samples (n = 336) with *Symbiodinium*-specific primers [62], using 0.1–1.0 µl of DNA, 2 µl 10x PCR buffer (Invitrogen), 1.0 µl 50 mM MgCl2, 1 µl 10 mM dNTPs, 1 µl 10 mM ITSintfor2, 1 µl 10 mM ITS2Clamp, 0.10 µl of Platinum *Taq* DNA Polymerase (Invitrogen) and dH_2_0 water to a total volume of 20 µl per reaction following LaJeunesse [62]. The amplified ITS2 fragments were separated using Denaturing Gradient Gel Electrophoresis (DGGE) on a Biorad DCode System following conditions outlined in Sampayo et al. [48]. Representative, dominant bands of each characteristic profile were excised, eluted overnight in dH2O, re-amplified using the non-GC primers [48], [62], and purified (using ethanol and ammonium acetate) prior to sequencing. The re-amplified PCR products were sequenced in both the forward and reverse directions (ABI BigDye Terminator chemistry, Australian Genome Research Facility). All chromatograms were aligned using Codoncode Aligner, checked manually and blasted on Genbank (http://www.ncbi.nlm.nih.gov/BLAST/). Maximum parsimony (MP) analysis was run in PAUP* 4.0b10 [82] under the delayed transition option and using indels as a fifth character state. All bootstrap values were calculated based on 1000 replicates.

### Statistical analyses

Dependence of genetic population structure on habitat and location was assessed for the host (mtDNA) and symbiont (ITS2) in a nested analysis of similarity (Two-way ANOSIM) using Bray-Curtis distance. Pairwise comparisons for each habitat were then performed in a One-Way ANOSIM to test for differences between individual habitats. Associations between host genotypes (mtDNA) and *Symbiodinium* types (ITS) were also evaluated using a One-Way ANOSIM. All multivariate statistics were done using the software package PRIMER (v6) [89].

## Supporting Information

Figure S1 Denaturing gradient gel electrophoresis of *Symbiodinium* ITS2 rDNA showing the 5 distinct *Symbiodinium* types found in *S. hystrix*: C120, C120a, C3n-t, C3-ff, C1m-aa. Characteristic sequences used to identify each symbiont type are shown adjacent to bands in the gel image (note that C3 and C3n co-migrate to the same position).
(1.71 MB TIF)

Table S1 Log probabilities L(K) and L' (K) for the likely number of genetic clusters in the microsatellite dataset, using STRUCTURE.
(0.05 MB DOC)

Table S2 Descriptive statistics for three microsatellite loci for *Seriatopora hystrix* collected from three habitats at two locations (7 populations). The last population consists of the individuals from the ‘Deep Slope’ habitat at Yonge Reef with a ‘HostD2’ mtDNA genotype.
(0.07 MB DOC)
