# Supplementary figures and images for: Genetic Divergence across Habitats in the Widespread Coral Seriatopora hystrix and Its Associated Symbiodinium
Source: PLoS One. 2010 May 27;5(5):e10871. doi: 10.1371/journal.pone.0010871 (PMC2877717; doi:10.1371/journal.pone.0010871)

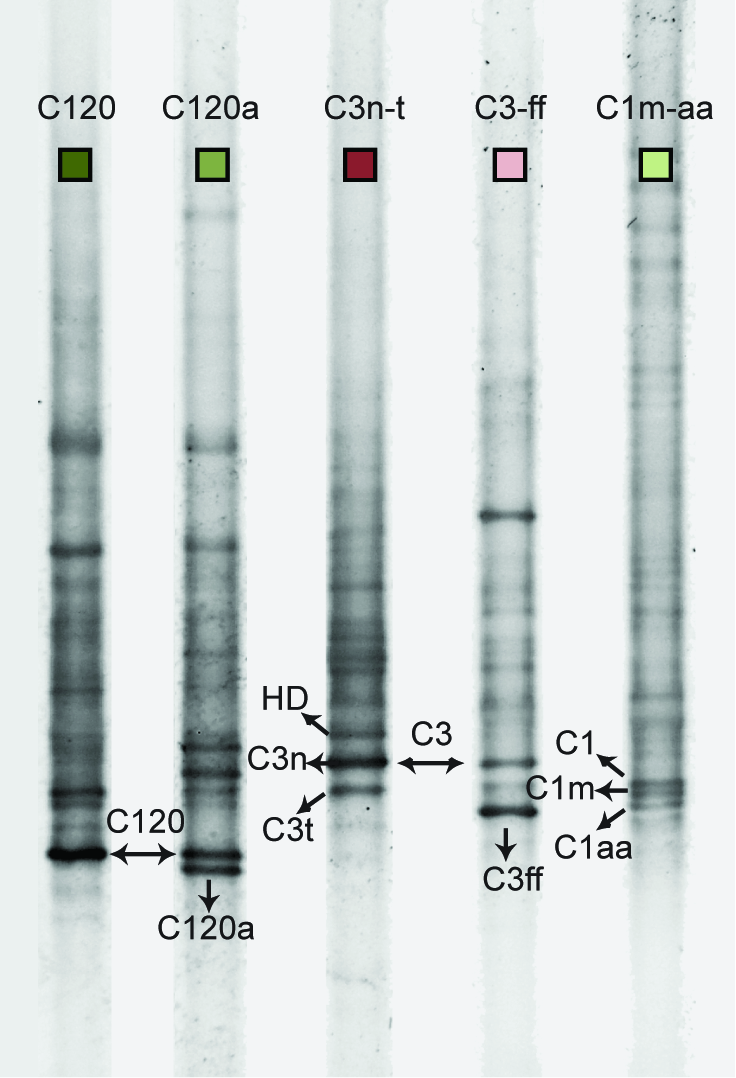

Supplement: Figure S1 — Denaturing gradient gel electrophoresis of Symbiodinium ITS2 rDNA showing the 5 distinct Symbiodinium types found in S. hystrix: C120, C120a, C3n-t, C3-ff, C1m-aa. Characteristic sequences used to identify each symbiont type are shown adjacent to bands in the gel image (note that C3 and C3n co-migrate to the same position). (1.71 MB TIF) [file pone.0010871.s001.tif]
