# Supplementary material for: Genetic Divergence across Habitats in the Widespread Coral Seriatopora hystrix and Its Associated Symbiodinium
Source: PLoS One. 2010 May 27;5(5):e10871. doi: 10.1371/journal.pone.0010871 (PMC2877717; doi:10.1371/journal.pone.0010871)
Supplement: Table S1 — Log probabilities L(K) and L' (K) for the likely number of genetic clusters in the microsatellite dataset, using STRUCTURE. (0.05 MB DOC) [file pone.0010871.s002.doc]

**Table S1** – Log probabilities L(K) and L’(K) for the likely number of genetic clusters in the microsatellite dataset, using STRUCTURE.

|  | **Including clonal genotypes** | | **Excluding clonal genotypes** | |
| --- | --- | --- | --- | --- |
| **K** | **L(K)** | **L'(K)** | **L(K)** | **L'(K)** |
| **1** | -2743.68 |  | -2094.1 |  |
| **2** | -2423.58 | 320.1 | -1863.96 | 230.14 |
| **3** | -2179.38 | 244.2 | -1682.1 | 181.86 |
| **4** | -2161.2 | 18.18 | -1658.8 | 23.3 |
| **5** | -2207.52 | -46.32 | -1707.56 | -48.76 |
| **6** | -2245.02 | -37.5 | -1747.48 | -39.92 |
| **7** | -2291.62 | -46.6 | -1778.92 | -31.44 |
| **8** | -2338 | -46.38 | -1810.66 | -31.74 |
| **9** | -2386.1 | -48.1 | -1843.22 | -32.56 |
| **10** | -2427.86 | -41.76 | -1874 | -30.78 |
| **11** | -2469.78 | -41.92 | -1902.74 | -28.74 |
| **12** | -2511.66 | -41.88 | -1932.42 | -29.68 |
