# Supplementary material for: Genetic Divergence across Habitats in the Widespread Coral Seriatopora hystrix and Its Associated Symbiodinium
Source: PLoS One. 2010 May 27;5(5):e10871. doi: 10.1371/journal.pone.0010871 (PMC2877717; doi:10.1371/journal.pone.0010871)
Supplement: Table S2 — Descriptive statistics for three microsatellite loci for Seriatopora hystrix collected from three habitats at two locations (7 populations). The last population consists of the individuals from the ‘Deep Slope’ habitat at Yonge Reef with a ‘HostD2’ mtDNA genotype. (0.07 MB DOC) [file pone.0010871.s003.doc]

**Table S2.** Descriptive statistics for three microsatellite loci for *Seriatopora hystrix* collected from three habitats at two locations (7 populations). The last population consists of the individuals from the ‘*Deep Slope*’ habitat at Yonge Reef with a ‘HostD2’ mtDNA genotype.

| **Population** | **Locus** | **Sh4-001** | **Sh2-002** | **Sh2-006** | **Across loci** |
| --- | --- | --- | --- | --- | --- |
| **‘*Back Reef*’** | N | 12 | 12 | 12 | 12 |
| **Day Reef** | A | 2 | 5 | 3 |  |
|  | H_E_ | 0.469 | 0.517 | 0.656 |  |
|  | H­_O_ | 0.250 | 0.500 | 0.833 |  |
|  | F_IS_ | 0.5000 | 0.0769 | -0.2291 | 0.0793 |
| **‘*Back Reef*’** | N | 46 | 46 | 46 | 46 |
| **Yonge Reef** | A | 4 | 8 | 7 |  |
|  | H_E_ | 0.631 | 0.835 | 0.703 |  |
|  | H­_O_ | 0.630 | 0.783 | 0.761 |  |
|  | F_IS_ | 0.0114 | 0.0740 | -0.0711 | 0.0088 |
| **‘*Upper Slope*’** | N | 20 | 20 | 20 | 20 |
| **Day Reef** | A | 3 | 4 | 5 |  |
|  | H_E_ | 0.541 | 0.554 | 0.776 |  |
|  | H­_O_ | 0.450 | 0.650 | 0.750 |  |
|  | F_IS_ | 0.1934 | -0.1488 | 0.0594 | 0.0370 |
| **‘*Upper Slope*’** | N | 31 | 31 | 31 | 31 |
| **Yonge Reef** | A | 5 | 6 | 6 |  |
|  | H_E_ | 0.595 | 0.745 | 0.709 |  |
|  | H­_O_ | 0.710 | 0.516 | 0.581 |  |
|  | F_IS_ | -0.1775 | 0.3216 | 0.1970 | 0.1342 |
| **‘*Deep Slope*’** | N | 21 | 21 | 21 | 21 |
| **Day Reef** | A | 3 | 6 | 6 |  |
|  | H_E_ | 0.482 | 0.594 | 0.630 |  |
|  | H­_O_ | 0.524 | 0.619 | 0.524 |  |
|  | F_IS_ | -0.0628 | -0.0176 | 0.1927 | 0.0476 |
| **‘*Deep Slope*’** | N | 19 | 19 | 19 | 19 |
| **Yonge Reef** | A | 4 | 4 | 11 |  |
|  | H_E_ | 0.619 | 0.571 | 0.848 |  |
|  | H­_O_ | 0.684 | 0.579 | 0.842 |  |
|  | F_IS_ | -0.0783 | 0.0125 | 0.0336 | -0.0063 |
| **‘*Deep Slope*’** | N | 15 | 15 | 15 | 15 |
| **Yonge Reef** | A | 3 | 8 | 10 |  |
| with ‘HostD2’ | H_E_ | 0.287 | 0.829 | 0.740 |  |
| haplotype | H­_O_ | 0.829 | 0.933 | 0.600 |  |
|  | F_IS_ | 0.1040 | -0.0919 | 0.2222 | 0.0644 |

N = number of samples per locus and location, A = number of alleles, H_E_ = expected heterozygosity, H_O_ = observed heterozygosity and F_IS_ = inbreeding coefficient.
